# The Israeli National Committee for sex selection by pre-implantation genetic diagnosis: a novel approach (2005–2011)

**DOI:** 10.1186/2045-4015-3-33

**Published:** 2014-10-28

**Authors:** Nirit Pessach, Saralee Glasser, Varda Soskolne, Amihai Barash, Liat Lerner-Geva

**Affiliations:** The National Social Work Service, Ministry of Health, 15 Noah Mozes St, Tel-Aviv, 67442 Israel; Gertner Institute for Epidemiology and Health Policy Research Ltd, Women and Children’s Health Research Unit, Tel Hashomer, 52621 Israel; The Louis and Gabi Weisfeld School of Social Work, Bar-Ilan University, Ramat-Gan, 52900 Israel; Department of Obstetrics & Gynecology, Kaplan Medical Center, Pasternak St, P.O.B 1, Rehovot, 76100 Israel

**Keywords:** Pre-implantation genetic diagnosis, PGD, Israel, Policy, Sex-selection, Assisted-reproductive technology, Assisted human reproduction

## Abstract

**Background:**

Pre-implantation genetic diagnosis (PGD) for fetal sex selection raises complex dilemmas. In Israel, PGD is regulated by the Ministry of Health. It is basically prohibited, but exceptions can be made upon approval by the National Committee for Sex Selection by PGD for Non-Medical Reasons (the “Committee”). This report describes the Committee’s work since its inception in May, 2005 through December, 2011.

**Methods:**

Files were abstracted onto a structured form. Discrete variables were analyzed by chi-square analysis, and continuous variables by T-Test.

**Results:**

During the study period 411 applications were received. Two-thirds of the applicants (n = 276; 67.2%) were Jewish and 26.8% were Moslem Arab. Over two-thirds (n = 285; 69.3%) had no children of the requested sex and ≥4 children of the opposite sex. Three-quarters of the requests were for a male (n = 308; 74.9%): 100% of Arab and 63% of Jewish applicants. Many noted more than one reason for their request. The most frequent category (n = 201; 48.9%) was a strong emotional desire, followed by medically-related reasons (n = 83; 20.2%).

For 216 applications a decision was arrived at, with 46 (21.3%) approved. Of the remaining 195 for 192 over a year had passed since last contact with the Committee. The likelihood of approval was higher if applicants met the criterion of ≥4 same-sex children than if they didn’t (33.7% vs. 11.6%, P = 0.001). The largest number of approvals were those requested for ‘emotional’ reasons, while the highest approval rate was for religious reasons.

**Conclusions:**

This study reviewed the first seven years of Committee activity. Most requested males, and the primary reason was the parents' intense emotional desire. Only one-fifth of the decisions were approvals, possibly reflecting reluctance to encourage non-medically-indicated PGD, a viewpoint not unique to Israel. Limitations include the relatively small number of cases and lack of access to Committee deliberation protocols. It is recommended that longitudinal studies be conducted to gain insight into the consequences to individuals, couples and families--both those whose requests were approved and those denied-- of this major step in reproductive technologies and in society’s effort to respond to them.

## Background

The use of pre-implantation genetic diagnosis (PGD) for the purpose of fetal sex selection raises complex ethical, social and professional dilemmas at the forefront of public debate throughout the world. While there is a general consensus justifying the use of the procedure for medical reasons, mainly to prevent the transfer of sex-linked genetic diseases, selection of the child’s sex for non-medical reasons (such as religious, financial, emotional, and/or family sex-balance considerations) is highly controversial [1, 2].

In many Western countries legislation prohibits the use of PGD for sex selection under all circumstances (e.g., Austria, Germany and Switzerland), or limits its use to medical purposes (e.g., Britain, France, Norway, Spain, New Zealand, India). In the United States, in contrast, the use of this procedure is not prohibited by law, and couples who are interested can freely approach a specialist clinic [3].

### Israeli policy regarding PGD sex-selection

In Israel, non-medical sex selection by PGD has been regulated since 2005 in a manner different from that in other countries [4]. The regulation of PGD is based not on legislation; instead it is mandated by a directive of the Ministry of Health [5]. The directive is guided by the premise that sex selection for non-medical reasons is basically prohibited, but that exceptions can be made in “highly unusual, irregular and rare cases” and after written permission is granted by the National Committee for Sex Selection by PGD for Non-Medical Reasons (heretofore referred to as the “Committee”). This Committee is appointed by the Director General of the Ministry of Health and is comprised of at least seven members including: an expert in medical/bio-ethics, a clinical psychologist, a social worker, a legal expert, a physician with expertise in genetics, a physician with expertise in obstetrics and gynecology who works in the field of fertility, an ethicist, and a clergyman (of the applicant’s faith). Committee members are selected from among the leaders in their fields in Israel, and few personnel changes have been made since the Committee’s inception. Legally married couples, common-law couples and single women can apply to the Committee, which can approve the procedure only if all of the following conditions are met:

1. There is real and imminent risk of significant damage to the mental health of one or both parents, or to the expected child, if the procedure is not conducted.
2. The applicants are married and have four joint children of the same sex and none of the other, except in extremely rare and idiosyncratic cases.
3. The applicants have received genetic counseling and information regarding all details of the PGD process, including chances of success, ethical considerations, with particular attention to the status and fate of the embryos of the non-selected sex.
4. Applicants clearly understand that if healthy embryos of the non-selected sex remain, permission will not be granted for additional in-vitro fertilization (IVF) cycles for sex selection until the remaining healthy embryos have all been used by the couple for reproductive purposes [4, 6].
5. Both parents have given informed written consent.

The Committee is authorized to consider applications with reference to “possible risks of in-vitro fertilization (IVF), the need for IVF and PGD for other reasons, and the familial and social situation of the applicants, including their age” [5]. Expenses incurred for required procedures are borne by the family, as IVF/PGD for non-medical reasons is not covered by National Health Insurance. According to the Directive, the protocols of the Committee meetings are confidential.

To date, no comprehensive study has been conducted regarding implementation of Israel’s current policy. In addition to presenting Israel’s ‘novel’ approach to dealing with the issue of sex-selection by PGD for non-medical reasons, the current report will describe the Committee’s work from the onset of its activity in May, 2005 through December, 2011, including the application procedure, characteristics of the applicants, reasons for request, aspects of the decision-making process, and the extent to which various types of requests are approved.

## Methods

### Committee procedure

**Figure 1:**
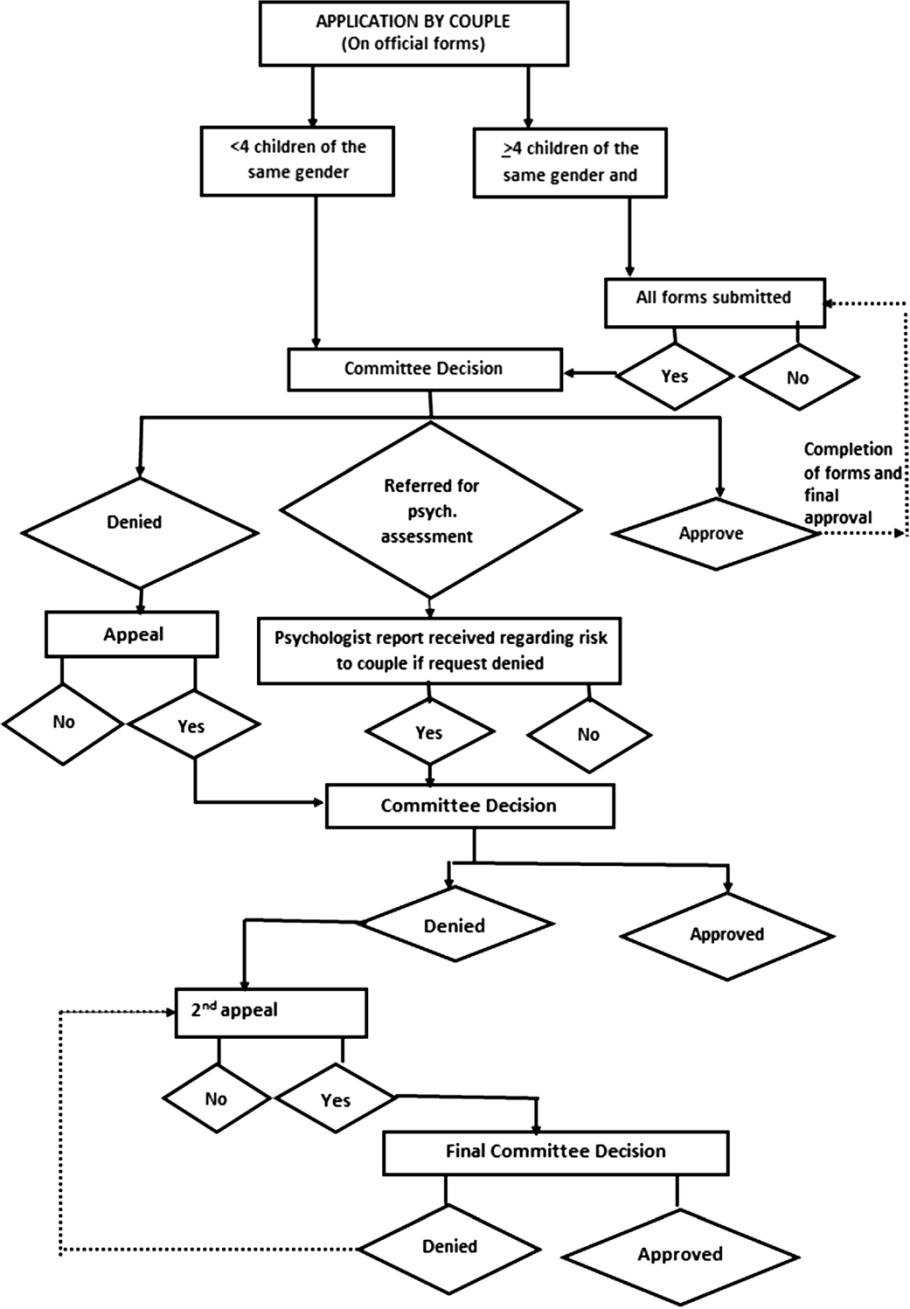
**Application and process of request for PGD for non-medical sex selection.**

The application procedure is described in Figure 1. Applicants to the Committee must explain their request on an official form, submit required medical documents, and undergo psychological assessment. Application forms are available to the public on the Ministry of Health’s website, and the Committee reviews all applications. All applications meeting the primary criterion of the couple’s having ≥4 children of the same sex and none of the opposite sex, as well as those exceptions deemed acceptable, are referred for psychological assessment and completion of additional medical forms. At a second Committee meeting each applicant’s file is presented. The file includes: (a) the original application; (b) a personal letter of request, explaining the motivation for the application; (c) medical documents regarding the couple’s health status and ob/gyn history; (d) a psychological assessment report. The psychological assessment is intended to evaluate the the extent of risk to the emotional well-being to either one of the partners and/or to their relationship, or to their family in the event that their request is denied. The assessment includes a clinical interview with each parent separately and with the couple together, and completion of a battery of objective and projective psychological tests (e.g., Rorschach Ink Blot Test, Dyadic Assessment Scale). The couple is referred to the psychologist only after the initial forms (application, medical, etc.) are completed and the request is not denied or approved outright. It should be stressed that the psychological assessment is a non-binding recommendation for the Committee’s consideration.

The Committee discusses and considers all relevant aspects before arriving at a decision. The applicants themselves do not appear before the Committee, with all communication (application, forms, response) conducted in writing. The factors considered by the members relate to the extent to which the application meets the directives for approval (as noted above). Final Committee decisions are made by vote, with a simple majority necessary. In its answers to the applicants, the Committee presents its decision with respect to whether the applicants meet (or do not meet) the criteria for approval, and/or whether the circumstances are “highly unusual, irregular or rare” to enable permitting (or denying) PGD, and thus there is serious justification for allowing (or denying) PGD sex-selection for non-medical reasons in their case.

Those whose request is denied may appeal and receive a second hearing, to defend or expand upon their request, either in person or in writing. The appeal is brought before the same Committee, with no change of membership.

### Committee record abstract

All application files and data from Committee meetings were abstracted onto a structured form specifically prepared for this study, and then coded and entered onto an Excel file. The free-text section on the application forms, in which the reasons for the request was stated, were reviewed by several researchers, in order to better define the various types of reasons. A consensus of categorization of the reasons was arrived at. The main categories included: emotional, medically-related, family/social pressure, religious and mental health. The distinction between the categories “emotional” and “mental health” was defined as follows: the former reflects the applicants’ emphatic expression of their desire for a child of the requested sex, while the latter refers to a reported or diagnosed mental health disorder. Regarding the ‘medically-related’ category, although the Committee deals only with non-medically-related PGD requests, some applicants noted reasons which could be construed as medically-related (e.g., maternal age, repeated Cesearean deliveries), although not sex-linked. More detailed descriptions of these categories are presented in Table 1. The psychological assessment was summarized by the Committee’s social worker (NP) from the report received by the Committee. To assure anonymity, on all forms each application was identified by a serial number only.

**Table 1:** **Reasons for requesting PGD sex selection**

Category (n*)	Detail
Emotional (509)	Intense desire and fulfillment of a dream
	Threat to family cohesion
	Wish to balance family
	For the benefit of others, incl. other children in the family
	Unfounded fear of sex-related genetic problem
	Response to a family tragedy
	Consideration of sex-selection by abortion or overseas PGD
Medically-related (192)	Final chance at pregnancy (maternal age, repeated CS)
	Non-sex linked genetic problem
Family/Social Pressure (172)	Continuing the family name
	Threat to the parent’s social status
	Dying request/will
	Financial implications
Religious (103)	Wish to fulfill the commandment to be “fruitful and multiply”
	To have a son to say *Kaddish* (memorial prayer, only by males)
	To spare the son of a *Kohen* public embarrassment of being born of sperm donation (i.e. request for a girl)
Mental Health: self-reported or diagnosed emotional/mental disturbance (30)	Anxiety
	Depression
	Obsession
	Psychiatric diagnosis
	Disturbance to functioning
	Avoidance of socializing

*Many applications noted more than one reason, even within categories; the number in parentheses refers to the total number of times that the reason was mentioned in the applications.

### Data analysis

Data was converted to the 8.2 release of SAS PC for analysis. Discrete variables were analyzed by chi square analysis, and continuous variables by T-Test, with a P value of 0.05 considered significant.

### Ethics

This research was approved by the Ministry of Health’s National Committee for Human Medical Research IRB (Helsinki Committee) (Approval No. 081–2011).

## Results

**Figure 2:**
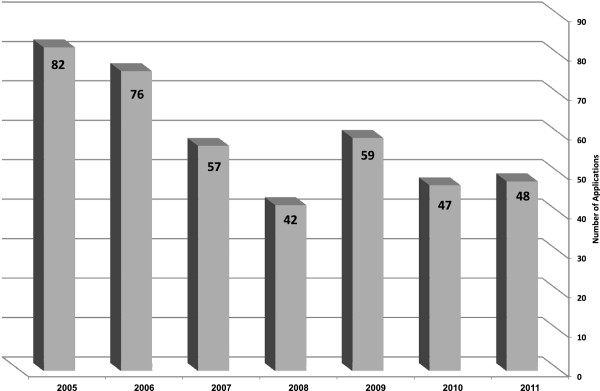
**Number of yearly applications to the committee (N = 411).**

Since its inception in 2005 and through 2011, the Committee met 47 times (approximately seven times yearly), and reviewed 411 applications. Although the number of applications varied from year to year (Figure 2), in general a drop is noted from 82 in 2005 to 48 in 2011 (mean: 59 per year).

### Demographic characteristics

#### Age

The mean age of the female and male applicants was 36.7 years (SD 4.4; range 21–48) and 40.6 years (SD 5.4; range 25–58), respectively.

#### Ethnicity

Two-thirds of the applicants (n = 276; 67.2%) were Jewish, and the majority of the others were Moslem Arab (n = 110; 26.8%), with six applicants Christian Arabs, ten Arab of undefined religion, and nine Druze couples. For further analyses ethnic group was dichotomized into Jewish and Arab (i.e. non-Jewish).

#### Marital status

The couples were all married, except for two couples who were in a stable partnership and stated their intention to marry.

#### Family composition

Over two-thirds of the applicants (n = 285; 69.3%) met the criterion of having at least four joint children of the same sex and none of the opposite sex. Of these, the majority (n = 233; 81.8%) had only girls. Over one-quarter of the applicants (n = 115; 28.0%) had fewer than four children of a single sex and none of the other, while 11 applicants (2.7%) had children of both sexes.

### Fertililty treatments

Since the PGD procedure involves IVF, a process which has inherent risks to maternal health, it is a consideration in the decision-making process. In cases where IVF would be necessary to achieve a pregnancy in any event, the Committee might consider this a factor influencing leniency in its decision. In 72 cases (17.5%), IVF was indicated independently of the sex selection request, and in 17 cases (4.1%) other fertility treatments would be necessary to achieve a pregnancy. In other words, in the remaining 78.4% of the applications the women would have to undergo an unnecessary IVF procedure to achieve the preferred-sex child.

### Preferred sex

Three-quarters of the requests were for a male child (n = 308; 74.9%). The preference for a male child was stronger among Arab applicants, with 100% so requesting, while among the Jewish applicants, over one-third (37.0%) requested selecting females (P <0.0001). Nearly all of those who requested a male child had no sons; however eleven couples who already had one or two sons requested another; mainly because the existing son(s) was handicapped in some way.

### Reasons for request

The reasons for applicants’ requests to the Committee and the number of times that each category was requested are presented in Table 1. Many applicants noted more than one reason for their request. Considering the only reason, or the first reason noted (when more than one reason was noted) on the 411 applications, the most frequent category in nearly half of the requests (n = 201; 48.9%) was the strong emotional desire. Second in frequency was the medically-related reason, noted in one-fifth of the requests (n = 83; 20.2%), and in descending frequency were family/social pressure (n = 60; 14.6%), religious reasons (n = 43; 10.5%), and mental health (n = 8; 1.9%). In sixteen applications (3.9%) no reason was specified on the application and this information was gleaned from the psychologist’s report.

### Psychological assessment

During the period under consideration, 136 couples were referred by the Committee to the psychologist; couples whose request was rejected outright were not referred to the psychologist. Of those referred (n = 136), almost all (n = 132) met the criterion of four children of the same sex. Only eighty-eight of these couples actually met with the psychologist and their reports were received, while the rest did not follow-up on the referral. Of those for whom the psychologist’s report was completed, nearly half (n = 43; 48.9%) were assessed to be at no clear risk in the event that their request was denied, while for the rest a significant risk was noted, and in some cases even multiple risks were noted. The risks were primarily to the wife’s emotional well-being (n = 30, 34.1%) or to the couple’s relationship (n = 23.1%; n = 26). The other risks noted were to the husband (n = 9), to both of them (n = 8) or to their children (n = 8).

### Committee application status

As of December 31, 2011, 411 applications were received by the Committee. Of these, 253 couples (61.6%) completed the forms and a first decision was arrived at: 14 couples were approved, 103 were rejected, and all of the remaining 136 couples were referred for psychological assessment. For 216 couples (52.6%) a final decision was arrived at. Of the remaining 195 applications, for 192 over a year had passed since last contact with the Committee, leading to the assumption that the couple had decided not to pursue the application process. The other three couples had not completed the process but less than a year had passed until the defining study end date of December 31, 2011.

### Committee decision

**Figure 3:**
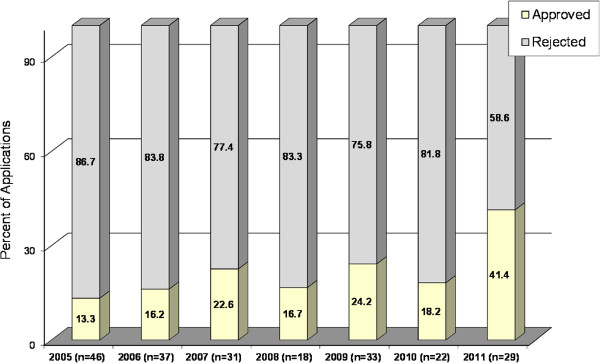
**Committee decisions by year of application (N = 216).**

The rate of approval rose over the years, from 13.1% in 2005 to 41.4.% in 2011 (Figure 3). Of the 216 applications for which Committee decisions were made, 46 (21.3%) were approved, and 170 (78.7%) were denied. The likelihood of approval was significantly higher if the applicants met the criterion of four or more same-sex children than if not (33.7% vs. 11.6%, P = 0.001). The rate of approvals to Arab couples was somewhat higher than that to Jewish couples (28.8% vs. 18.5%) although this trend did not reach statistical significance. Regarding the eleven couples who had one or two sons and requested a son, only one of these was approved, and the others were rejected.

### Appeals of committee decision

Fifty-two (30.6%) of the 170 requests denied by the Committee were appealed. Eleven of these appeals were granted; the rest were denied.

### Committee approval criteria

Thirty-two of the 46 applications approved met the primary criterion of having four or more children of the same sex and none of the requested sex. Thirty-one of these were in response to requests for a male child, and only one for a female. Two of the remaining applications were approved for “rare idiosyncratic circumstances”, and twelve were approved on the basis of a consideration unique to Jewish families--the case of fathers who are *Kohanim*, i.e. of the priestly families [4]. In these cases the special status of *Kohen* is passed from father to son(s), and when the son reaches maturity, at the age of 13, he can join in offering the priestly blessing in the synagogue. Since this is an obviously public practice, some *Kohanim,* whose wives could become pregnant only by sperm donation, requested that only daughters be born to them, thus sparing the potential serious emotional injury to a son who would not be his genetic offspring. In two of these cases, two PGD procedures were approved.

Regarding the decisions following appeal, ten of the eleven families approved (90%) met the criterion of four children of a single sex and none of the requested sex. Nevertheless, it should be noted that nearly two-thirds of the rejected appeals met this criterion.

### Committee decision by reason for request

The rate of approval as related to the reason for the request is presented in Table 2. The largest number of applications received was requested for ‘emotional’ reasons (see Table 1 for details), while the highest rate of approval was for religious reasons, both for *Kohanim* and for non-*Kohanim*.

**Table 2:** **Committee approval by reason for request**

Reason*	Distribution of reasons among applications decided upon*	Rate of approval by reason*
	N (%)	n (%)
Total Applications Decided upon:	216 (100.0)	46 (21.3)
Emotional	99 (45.8)	18 (18.2)
Medically-related	48 (22.2)	5 (10.4)
Family/Social Pressure	29 (13.4)	5 (17.2)
Religious--*Kohen*	11 (5.1)	11 (100.0)
Religious--Not *Kohen*	16 (7.4)	5 (31.3)
Mental Health: self-reported or diagnosed emotional/mental disturbance (30)	9 (4.1)	2 (22.2)
No reason specified	4 (1.9)	0

*Number of applications offering the particular reason alone, or as the first reason noted on the application, in cases where more than one reason was given.

## Discussion

The present study reviewed the first seven years of activity of Israel’s National Committee for Sex Selection by Pre-Implantation Genetic Diagnosis. The study’s aims were to present Israel’s “novel” approach to the issue, to describe the Committee’s work during its first seven years, including procedures, characteristics of applicants, applications, and decisions, as well as aspects of the decision-making process. A majority of the couples applying and a majority of those approved met the primary criterion of having four joint children of one sex and none of the requested sex. Most of the applicants requested male children, and the primary reason for this request was the parents’ intense emotional desire, although often more than one reason was given.

Interestingly, while the number of applications dropped over the years under consideration, the rate of approvals rose. Regarding the drop in applications, one can conjecture that families who may have considered applying heard from others of the high rate of rejections, thus avoided doing so. Following the number of applications over the coming years may confirm whether this is a stable trend, but clarification of this question would require a different type of study.

The Committee completed deliberations on about half of the 411 applications submitted during the study period, and one-fifth of these were approved. While a rise in the rate of approvals is noted in 2011, the numbers are small, so it is as yet unclear if this trend will continue or is sporadic, and/or whether this reflects a less stringent attitude on the part of the Committee. However, even among those who met the main criterion of having four same-sex children and none of the requested sex, only one-third were approved. This finding may well reflect a general reluctance on the part of the Committee members--who come from several disciplines--to encourage non-medically-indicated PGD. This viewpoint is not unique to Israel.

Opponents of PGD for non-medical reasons claim that free use of medical technology for sex selection may lead to: (1) danger of upsetting the demographic balance between the sexes, as in India and China, where abortion of female fetuses has resulted in a lack of millions of women [7]; (2) danger of the “slippery slope” whereby fetal sex selection is the first step toward “custom made babies”, and unprecedented interference in the act of creation [2, 8]; (3) psychological harm to the “custom made child” who must withstand the pressure of meeting parental expectations, and concern for damage to other children in the family [9–11]; (4) discrimination against women by diminishing their very existence [8, 11]; (5) the inevitable destruction of pre-embryos [12, 13]; (6) medical risks to the mother and offspring as a result of technological methods employed [10, 11, 13] and (7) inappropriate and potentially unfair use of limited medical resources [13, 14].

On the other hand, proponents claim that fetal sex selection, particularly in non-Western cultures, enables control over the size of the population, eases economic burden and liberates women from the pressure of multiple pregnancies, or in worse cases aborting female fetuses and/or neglecting female babies [13, 15, 16]. Ethicists in the West support this position with different arguments, such as: (1) the right to reproductive autonomy; (2) the claim that Western countries are less vulnerable to the risk of imbalance between the sexes; or (3) the claim that a preference for sex balance in the family is not necessarily sexist [17–19].

Feminists have expressed mixed views regarding sex selection [20]. Whereas some believe it enhances the autonomy of women, others maintain it provides an additional manifestation of discrimination against them [7, 20].

Leading international professional organizations have acknowledged the complex implications of the use of PGD for non-medical reasons. Several organizations, such as WHO, UNESCO and HFEA (Human Fertilization and Embryology Authority, UK), have published policy statements opposing its use. However, ESHRE (European Society of Human Reproduction and Embryology) and ASRM (American Society for Reproductive Medicine) have expressed divergent views on the issue, and note that the use of PGD for social reasons would be reasonable under certain circumstances, notably with an emphasis on family sex-balancing [3, 21]. ESHRE’s (2013), recent recommendations leave room for re-evaluation of blanket rulings permitting or banning non-medical PGD sex-selection, for example, in cases of those who must undergo IVF for medical reasons [21].

The above reflects some of the major issues involved in non-medical PGD, although in- depth discussion of the ethical aspects was not within the scope or purpose of this research. While they are complex and compelling, they have been and are continuing to be discussed in a broad range of publications [2–4, 21, 22].

In an attempt to evaluate possible demographic and social implications for the balance between sexes in the event of freer and greater use of existing technologies, many studies around the world deal with the identification and analysis of trends in favoring the sex of offspring [1, 18, 23–25] and note obstetric and socioeconomic background variables as being associated with it. Regarding the former, about one-fifth of the applicants in the present study would have had to undergo IVF for other medical reasons, and might have expected this to enable their eligibility, but this did not guarantee approval of PGD. Although it was beyond the scope of the present study to investigate the reason for dropouts, this may be due, at least in part, to the stress of the IVF procedure itself [26, 27]. Regarding socioeconomic factors these could be relevant due to the fact that, as opposed to medically-indicated PGD, the procedure for sex-selection purposes is not covered by the National Health Insurance Basket of Services in Israel, and thus couples would incur significant financial cost.

Family size and composition have been considered a major motivator for sex-selection. Many studies, including studies in Israel [28, 29], have indicated that in families where there are children of a single sex, and especially in cases of at least three of the same sex, there is clear preference for the other sex [1, 23–25, 30, 31] The association between family composition and preference for offspring’s sex is not surprising given the ongoing trend of declining total fertility rate in Western countries [32], as well as in Israel [33]. An Israeli study regarding attitudes towards PGD included a survey among a population of married couples of reproductive age who were parents to at least two children of only one sex [29]. In their sample it was found that 45% of the respondents supported permitting sex selection for non-medical reasons and 42.6% wished to select the sex of their own future child. However, many of those expressing opposition in principle were willing to allow this in cases of psychological or familial crisis.

In the present analysis, while having four children of the same sex and none of the opposite sex was a prerequisite for approval, nevertheless nearly one-third of the applicants did not meet this criterion. Although this was the primary prerequisite, it does not necessarily indicate that applicants disregarded the official directives. This is because the Directive clearly allowed for exceptions in cases judged by the Committee to be “extremely rare and idiosyncratic” [4, 5]. Certain families evidently felt that they met this condition, for example those who already had a son but he was handicapped, and they requested another son.

Among those who did not meet this requirement is the sub-group of *Kohanim* (see above), in which cases the Ministry of Health’s legal advisor reviewed the issue, and decided that leniency would be considered, due to the potential for ‘injury to the child’ and the likelihood that the couple would refrain from having children at all. This represents a unique situation in Israel and among other Jewish communities worldwide. Similarly, decisions based on other unique religious or cultural reasons may occur in other societies.

Sex preference for offspring is often grounded in religious and cultural traditions and in social norms which shape the attitudes and behavior of the individual [1]. Research has consistently indicated a dominant trend in Western societies favoring a mixed and balanced composition of the sexes in the family, when a preference exists at all [1, 19, 30, 34]. On the other hand, a preference for sons has been reported with respect to many religions [30, 35]. A clear preference for boys has been reported in Eastern Asian (China, India, Korea, the Philippines) [22] and in Moslem Arab societies, based on religious beliefs, rigid paternalistic traditions and primacy of the male social role [1, 18, 19, 22–25, 30, 31]. Notably, emigration to other cultures has not been found to change the cultural norms of emigrants who live in communities that preserve the values, traditions and cultural norms of their country of origin [22]. This preference is reflected in the current findings, in which all of the Arabs and almost two-thirds of the Jews (not including *Kohanim)* requested males. Details of the couples’ sociodemographic characteristics (e.g., educational level, income, religiosity) were not available for this study to clarify possible differences between the groups on these aspects, since they are not included in the application forms. It is possible that this indicates not simply the preference for male offspring, but the degree of effort, expense, etc. that those wanting sons are willing to expend in comparison to those wanting daughters. Interestingly, this is in contrast to the findings of Hashiloni-Dolev’s et al. Israeli survey [29], in which there was no significant difference in the rate parents of ‘only boys’ or ‘only girls’ stating that they would be interested in choosing the sex of their next child. This may reflect the distinction between a population sample and a specific cohort, as well as between a hypothetical question and actual personal experience.

Fertility issues have also been associated with sex preference of offspring. Parental age, particularly advanced maternal age and approaching menopause, has been related to a desire to proactively select the offspring’s sex [23, 24], Among the couples in this study, maternal age or having had repeated Cesarean section deliveries (making additional deliveries risky) were noted in almost half of the requests, and most of those who reached the Committee’s decision stage with this noted as their primary reason were rejected. Among fertility clinics in the U.S., the necessity for the woman to undergo IVF or PGD for non-sex-linked medical or genetic reasons has been found to be a factor that strengthens the preference and willingness to conduct additional PGD for sex selection [23]. However, a study conducted on couples undergoing fertility treatments in Germany found that 90% of respondents ruled out the possibility of using PGD for sex selection for non-medical reasons even if the technology would be accessible to all [36].

Another aspect of the dilemma involved in PGD sex-selection may be the emotional cost to the parties involved of promoting desire for a child of the requested sex by offering the option of choice. It is notable that almost thirty percent of the applicants did not complete the application process. When possibilities exist, they may open avenues of expectation, which may in turn lead to disappointment when these expectations are not fulfilled. Schwartz [37], considering consumer options, has called this the “Paradox of Choice”, stating that while autonomy and freedom of choice are important for well-being, Americans have more choice than ever, but do not seem to be benefiting from this psychologically. More specifically relating to reproduction, others have noted the irony inherent in the fact that new reproductive technological advances may become a source of stress by offering the option of choice. As Rothman stated: “The technology of prenatal diagnosis has changed and continues to change women’s experience of pregnancy” [38]. McQuillan discussed the irony inherent in new reproductive technologies that open options both for women dealing with childlessness, as well as for those who do not have fertility problems, concluding that “… these choices may be yet another source of stigma and stress for women who do not choose to pursue medical treatment or to pursue it to its extreme” [39]. The question remains as to whether the choice of sex-selection enables families to fulfill their hopes, or results in pressure on them--particularly on women--to meet expectations that have become available, or both.

Israeli PGD policy attempts to deal with the various issues involved. In the Introduction to the MOH Directive [5] the considerations on which the guidelines are based are detailed, as translated (SG): “Considering, on the one hand, the basic human freedom to choose for oneself when the technological means are available, and on the other hand, factors important for an orderly and moral society, among them medical and ethical reasons opposing unnecessary medical procedures that carry potential risk, status of embryos of the unwanted sex, preventing sex discrimination, and maintaining the demographic sex balance…in addition not ignoring the high financial cost of conducting PGD which requires IVF”. Despite the basic negative attitude towards non-medical PGD, the Directive attempts to provide a solution for exceptional cases in a way that reduces some of the personal, familial and social risks associated with the use of PGD technology for sex selection. The threshold criterion of at least four children of the same sex is intended to prevent the risk of imbalance between the sexes in society (the male-to-female ratio in Israel in 2011 was 1.05 [33]. The requirement that both spouses sign a consent form is meant to ensure that the applicants fully understand the procedure and its implications for the health of the mother and fetus. The restriction requiring the use of any remaining embryos before permitting another IVF cycle is intended to: (a) reduce potential ethical problems regarding the status of unwanted embryos, and (b) avoid potential injury to the woman’s health due to unnecessary medical procedures [5].

Several authors have addressed this unique Israeli policy. While the policy is designed to allow a degree of flexibility in Committee judgments, some criticize the lack of any absolute criteria, even that of the four-children-of-only-one-sex criterion [4, 10]. Some justify or refute it from a religious-*Halachic* (Jewish religious law) point of view [16, 40, 41]; some believe that allowing PGD could avoid abortions of fetuses of the undesired sex [42, 43], which are illegal in Israel on such grounds. Some call for legislative regulation of the issue [12, 41]. Others have vehemently opposed this policy for various reasons, mainly impingement on individual freedom, the principles of the liberal-democratic state [44], danger to the health of the mother and the emotional health of the future child and his siblings who were born “randomly”, [9] and danger to intra-familial relations [10].

The Israeli policy is presented here as one of the possible resolutions to the complex issue at hand. In light of the broad differences between countries and their cultures, the Israeli model may serve as one among several models for consideration. While it might not be appropriate for adoption, it may offer a basis from which elements could be adapted.

## Conclusion

The present study reviewed the first seven years’ activity of the Committee for Sex Selection by Pre-implantation Genetic Diagnosis. Israeli policy toward PGD for non-medical reasons is basically prohibitive. However it is unique in allowing for exceptional permissions on an individual basis, enabling a degree of flexibility in Committee judgments. Only one-fifth of the Committee’s decisions were to approve the procedure. A majority of couples applying and a majority of those approved met the primary criterion of having four joint children of one sex and none of the requested sex. Most applicants requested a male child, and the primary reason for this request was the parents’ intense emotional desire, although often more than one reason was given.

Although this study included all applications to the Committee from its inception, the relatively small number of cases limits the ability to conduct in-depth data analysis. While there is definitely import to a preliminary view of the Committee’s activities, many questions remain unanswered; dilemmas unresolved. Although the present investigation offers previously unavailable insight into many aspects of the Committee’s work, details of the deliberations and protocols were not available to the investigators. Future research may be able to access this information, which could enlighten understanding of the Committee’s considerations. It is also recommended that the scope of this research be expanded upon as the database grows (i.e. more applications), so that greater understanding can be gained into the dynamics of the Committee procedures, as well as possible implications on the larger society of “social” sex-selection. Further, as more couples apply for Committee approval for non-medically-indicated sex-selection by PGD, it is strongly recommended that qualitative and quantitative longitudinal studies be conducted to gain insight and understanding into the personal consequences to individuals, couples and families--those whose requests are approved, those whose are denied, and those who drop out after beginning the application process--of this major step in twenty-first century fertility technologies and in society’s effort to respond to them.

## Authors’ information

NP is former Head of the Department of Social Work in General Health Care, Ministry of Health Social Services Department, currently doctoral student at the Weisfeld School of Social Work, Bar-Ilan University; SG is a Developmental & Health Psychologist and Senior Researcher at the Women and Children’s Health Research Unit of The Gertner Institute of Epidemiology and Health Policy Research; VS is Associate Professor at the Louis and Gabi Weisfeld School of Social Work, Bar-Ilan University, Deputy Head of the School and Chair of the Bachelor’s Degree Program; AB is former Director of the IVF Unit at Kaplan Medical Center and current Chairman of the Israeli National Committee for Sex Selection by Pre-implantation Genetic Diagnosis; LLG is the Director of the Women and Children’s Health Research Unit of The Gertner Institute of Epidemiology and Health Policy Research, and Associate Professor at the School of Public Health, Sackler Faculty of Medicine, Tel Aviv University.
